# Association of Covid-19 pandemic-related stress and depressive symptoms among international medical students

**DOI:** 10.1186/s12888-021-03671-8

**Published:** 2022-01-07

**Authors:** Lu Lu, Xiaobin Wang, Xuehang Wang, Xiaoxi Guo, Bochen Pan

**Affiliations:** 1 Center for Reproductive Medicine, Department of Obstetrics and Gynecology, Shengjing Hospital of China Medical University, No. 39, Huaxiang Road, Tiexi District, 110022 Shenyang, P.R. China; 2 China Medical University-The Queen’s University of Belfast Joint College, China Medical University, Shenyang, P.R. China; 3 International Education School, China Medical University, Shenyang, P.R. China

**Keywords:** Covid-19, Stress, Depressive symptoms, Coping style, Perceived social support

## Abstract

**Background:**

The outbreak of Covid-19 had negative impacts on the mental stress and induced psychological distress among university students worldwide. This study proposed a moderated mediation model, and hypothesized that the Covid-19 pandemic-related stress was positively related to depressive symptoms among international medical students.

**Methods:**

An online survey on stress and depressive symptoms of international students was conducted in a medical university. Questions on Covid-19 pandemic-related stress, Patient Health Quesionnaire-9, Simplified Coping Style Questionnaire and the Perceived Social Support Scale were used as measurements, and model analyses were conducted using Hayes’ PROCESS macro for SPSS.

**Results:**

It was found that 9.83%, 3.08% and 2.12% students had mild, moderate and severe depressive symptoms, respectively, and the positive association between Covid-19 pandemic-related stress and depressive symptoms was significant (β = 0.27, t = 6.87, P < 0.01). Negative coping was also significantly correlated to depressive symptoms (β = 0.26, t = 6.60, P < 0.01), and partially mediated the association between Covid-19 pandemic-related stress and depressive symptoms. Perceived social support had a negative association with depressive symptoms (β=-0.26, t=-6.25, P < 0.01), played a negative moderating role in the relationship between negative coping and depressive symptoms, and moderated the indirect effect of Covid-19 pandemic-related stress on depressive symptoms via negative coping.

**Conclusions:**

Results of the study suggested that under the background of continuing pandemic, intervention or prevention of mental health problem is urgently needed for the international students, and depression may be alleviated through reducing negative coping and increasing perceived social support.

## Background

There are a variety of stressful events that people have to face in their lives. If the intensity of stress exceeds the individual’s ability to deal with, it may cause psychological or physical damage to the individual [1]. The severe acute respiratory syndrome coronavirus 2, which has the characteristics of rapid transmission and high infection rate, was identified at the end of December 2019, but it took only a few months before it was declared as a global pandemic by the World Health Organization in 2020. Unfortunately, it is still rampant in some parts of the world and has negatively influenced daily activities and mental health of the general population [2, 3]. Such extremely serious event may be regarded as a serious threat (stressor) to the majority of people, especially the healthcare professionals at the early stage of the pandemic [4], as they were at greater risk of being infected and in a situation of demanding more moral responsibilities [5, 6]. On the other hand, evidence across the world indicated that the younger people including children were vulnerable to the mental health problems during the Covid-19 pandemic [7–9]. Indeed, most recent studies have revealed that the university students were suffered from more psychological stress and experienced more depressive and anxiety symptoms during the pandemic due to the lockdown, uncertainty and life disruption [10, 11]. Among them, medical students deserve special attention during the pandemic, as they are the future healthcare professionals, and the negative impact of Covid-19 may influence their future career choice [12]. Recent studies have confirmed that the Covid-19 pandemic had negative impacts on the mental stress and induced psychological distress among medical students [13]. Therefore, it’s crucial to take care of the mental health of medical students during the pandemic.

The number of international students in China has kept increasing since the 21st century, which has brought challenges to Chinese universities in providing good services especially health support to international students. International students are a special population of students in medical universities, but the studies on their psychological status are scarce, in particular during the pandemic. Therefore, the present study aims to help educators, administrators and students of higher educational institutions to manage the pandemic related stress by conducting a survey on the international medical students and analyzing the mechanism of interaction between the stress-related variables and the adverse mental health outcomes. For that purpose, we will put forward and test several hypotheses in this study. Our first hypothesis (H1) is: There is a positive association between Covid-19 pandemic-related stress and depressive symptoms among international medical students.

So far, there has no consistent research conclusion on the mechanism of psychological stress reaction induced by stressors. Lazarus and colleagues put forward a theory of cognitive appraisals and coping, which assumes that the individual’s appraisal of stressors influences his or her coping strategies and then affects the consequences of the stressors [14]. In this theory, coping is described as continuous cognitive and behavioral efforts to manage demands that exceed individuals’ resources [15]. Coping style refers to the coping strategy that an individual takes to protect himself or herself from the deleterious effects of stressors. According to this theory, individuals can adjust to the emotions activated by the stress through coping strategies, and manage the problems caused by the stress [16]. Given the importance in managing stress, coping has received much attention, and various models of coping have been developed. In spite of the type varieties, it has been widely accepted that coping is an important mediator between stress and psychological stress reactions.

Previous studies have revealed that coping mediated the association between stress and depression, and could predict the severity of depression. For example, Shen and colleagues explored the effect of stress on college students’ depression, and found that the positive and negative coping styles were partial mediators between stress and depression [17]. Xu and colleagues investigated the mediating role of coping in relationship between work stress and mental health of nuclear enterprise employees, and found that work stress not only posed a direct effect on mental health but also had an indirect effect on mental health through negative coping style [18]. In the study of depressive symptoms among overseas Chinese students conducted by Chou et al., it was suggested that passive coping strategies mediated the relation between stress and depressive symptoms [16]. Lardier Jr et al. examined the effect of coping style on well-being among Hispanic undergraduates, and found that higher level of stress was associated with higher level of reactive and suppressive coping style, which led to more subsequent depressive symptoms [14]. In addition, according to the similar research performed by Rogowska and colleagues during the Covid-19 pandemic, partial mediation effect of coping style was also found on the relationship between stress and life satisfaction among university students [19]. Based on these findings and the stress coping theory, we propose the second hypothesis (H2): Coping plays a mediating role in the association between Covid-19 pandemic-related stress and depressive symptoms among international medical students.

On the other hand, social support is an important factor considered beneficial to individual’s psychological health. It is defined as the psychological and/or physical assistance provided by family, friends and others to an individual facing difficulties [20]. A large body of research has focused on social support as a mechanism that protects people from the deleterious effects of stress. There are two hypotheses on the mechanism. One is the main-effect model, in which social support provides beneficial effects on mental health independent of stressors. For example, Aneshensel and Frerichs’s investigation presented that social support had a direct, positive impact on the individual’s psychological well-being irrespective of level of the stressors [21]. The other is the buffering model, which posits that social support alleviates the impact of stressors on mental health only in times of high stress. Cohen believed that social support may act on the link between stressful events and individuals’ appraisal, and play a moderating role in the relationship between the subjective experience and psychological damage [22]. However, Stroebe et al. proposes another way that social support may help individuals’ recovery from the stress reaction more easily [23]. In general, beneficial buffering effects of social support have been considered to enhance the individual’s sense of mastery in coping with stress and to diminish helpless feelings [24].

**Fig. 1:**
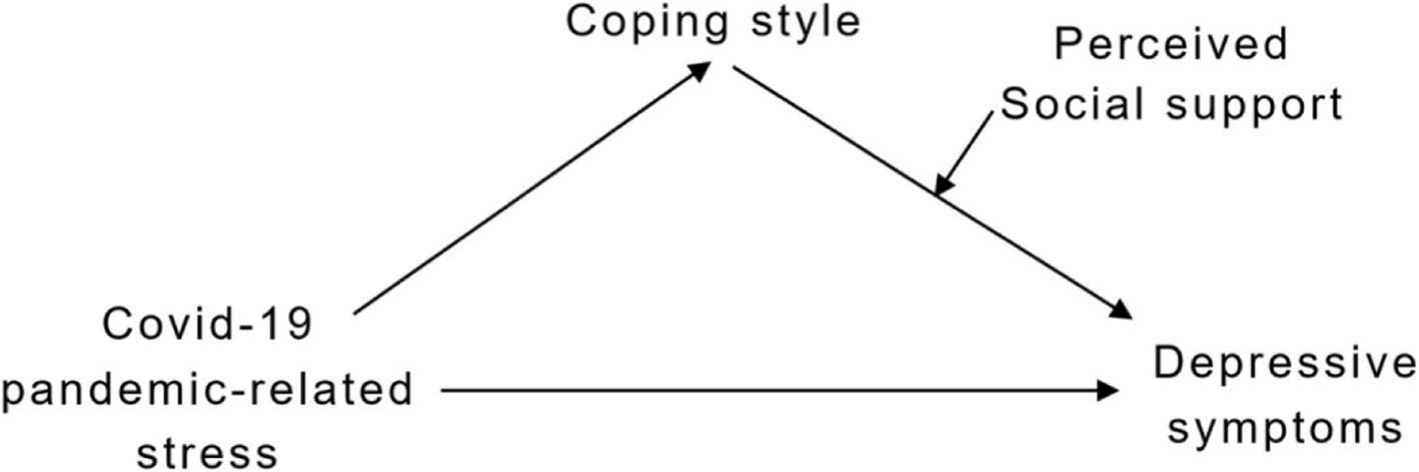
Conceptual framework of the study

Social support is most often categorized into two basic types: received social support and perceived social support. Received social support is the objective or actual support, including assistance of materials and direct services. Perceived social support refers to the cognitive perception of availability and adequacy of support from others [25]. Although social support theory emphasizes the role of actual support, most evidence shows that perceived social support plays the essential function, especially under the condition of stress [26]. In relation to depression, it is reported that perceived social support is an important predictor of depression [27] as well as a buffer of depression in response to life events and chronic strains [24]. In a four-year prospective model of cardiac patients, Holahan et al. found that the ongoing social support enhanced coping efforts, whereas the social stressors eroded coping efforts [28]. Thus, there might be a moderating effect of perceived social support on the relationship between coping and depression. Based on these findings, we further formulate the following hypotheses: (H3) Perceived social support plays a moderating role in the association between coping and depression among international medical students; (H4) Perceived social support moderates the indirect effect of Covid-19 pandemic-related stress on depression through coping. The conceptual framework of our study is summarized in Fig. 1.

## Methods

### Study design and subjects

A cross-sectional study, an online survey was conducted during November 2020. The target population for the study was the international students currently studying at China Medical University. In order to participate in the study, the students should meet the following inclusion criteria: (1) a current student of the University, (2) can get access to the internet, (3) can read and understand the survey content. The email addresses of 1030 potential participants who met the inclusion criteria were provided by the staff from International Education School. The procedures for the study began with emails to the potential participants which described the purpose of the study and a link to access the online questionnaire. When the participants visited the website to answer the survey, they were greeted with an informed consent letter stating that the survey was completely voluntary. In total, 550 international students gave their consent to participate, among whom 519 completed the survey with a response rate of 50.39%. The study had been approved by the Research Ethics Committee at China Medical University.

### Measurements

#### Measurement of depressive symptoms

Depressive symptoms were assessed with the Patient Health Questionnaire-9 (PHQ-9), which asked the frequency of problems that bothered the respondents. It consists of nine items with a four-point Likert scale from 0 “not at all” to 3 “nearly every day” [29]. The total score was used to determine the severity, where the higher scores indicated more severe depressive symptoms. A total score of 10 or higher indicates the presence of depressive symptoms, and previous studies have shown that the sensitivity and specificity were 88% and 88% respectively for detecting major depression [30, 31]. The total score can be further categorized as mild depressive symptoms (10-15), moderate depressive symptoms (16-21) and severe depressive symptoms (22-27) [30]. PHQ-9 has been proven to be a reliable measurement in the prior studies with Cronbach’s alpha being 0.86-0.89 [20]. In this study, Cronbach’s alpha was 0.90.

#### Measurement of Covid-19 pandemic-related stress

Covid-19 pandemic-related stress was measured by 5 questions regarding (1) the perception of Covid-19 outbreak, (2) worry about being infected, (3) worry about family/relatives/friends being infected, (4) worry about the exam scores and (5) worry about not being able to complete the study [32–35]. Participants answered 1 (not at all) to 4 (very much) to the questions. Higher total score represents higher level of Covid-19 pandemic-related stress. A Cronbach’s alpha of 0.80 was found in this study.

#### Measurement of coping

Coping was evaluated by the Simplified Coping Style Questionnaire (SCSQ), which was developed by Xie and Zhang based on the coping styles cognitive theories [36]. It consists of 20 items to identify the attitude or potential actions that an individual will adopt when experiencing setbacks or difficulties. Each item was rated on a four-point scale ranging from 0 (never) to 3 (very often). The dimensions of the questionnaire include positive coping style (12 items) and negative coping style (8 items). The positive coping style reflected positive coping strategies, such as “looking for suggestions from relatives, friends or classmates”. The negative coping style reflected negative coping strategies, such as “relying on somebody else to solve the problem”. Higher scores indicate preference of adopting the relevant coping style. The questionnaire has been proven to have a good reliability and validity in previous studies [37, 38]. The Cronbach’s alpha for this sample was 0.91.

#### Measurement of perceived social support

The Perceived Social Support Scale (PSSS), comprising of 12 items scored on a 7-point rating scale (1 = very strongly disagree, 7 = very strongly agree), was used to measure the perceptions of social support received from three sources: family, friends and others [39]. A higher summative score indicates a higher level of social support perceived by an individual. The scale has a good internal reliability with Cronbach’s alpha coefficient of 0.88 to 0.90 [40, 41]. The Cronbach’s alpha for PSSS in the present study was 0.94.

### Demographic characteristics

Demographic characteristics included age, gender, educational background, current place of residence (China/Asia outside China/Africa/Europe/North America/Oceania), residence style (Live with family or friends/ Live alone) and Covid-19 outbreak in the city (No/Yes/Do not know the actual situation).

### Statistical analysis

Descriptive statistics were calculated for obtaining detail information regarding each variable within the total sample. We tested the normality for continuous variables before data analysis and found the scores of PHQ-9 were not normal distribution. Mann-whitney U test and Kruskal-Wallis H test were conducted to determine whether significant differences existed between categorical variables. To examine correlations between continuous variables, Spearman’s rank correlation coefficients were used.

Statistical analyses were conducted using SPSS 17.0 and PROCESS macro program for SPSS. The PROCESS macro program was performed to run a regression-based path analysis, which could verify moderated mediation models and mediated moderation models based on the bias-corrected percentile bootstrap method. The hypothesis model was tested with 5000 resampled samples by estimating the 95% confidence interval for the mediation and moderation effects. The result was considered significant if the 95% confidence interval does not contain zero [42]. It was suggested to perform the testing from the simpler model to more complex one [43]. Therefore, we started with examining the simple mediation model of coping by Model 4 in PROCESS, which generated direct and indirect effects in mediation. In this model, Covid-19 pandemic-related stress was entered as X, PHQ-9 was entered as Y and coping style was entered as the mediation variable. When the direct effect became nonsignificant, but the indirect effect was significant, then full mediation was established. Partial mediation was confirmed if both effects are significant [44]. Once the simple mediation model was confirmed, the moderated mediation model of coping style and perceived social support was run using Model 14 in PROCESS. In this model, Covid-19 pandemic-related stress was entered as X, PHQ-9 was entered as Y, coping style was entered as the mediation variable and perceived social support was entered as the moderation variable in the association of coping style and PHQ-9. This model tested whether the direct effect or the indirect effect between Covid-19 pandemic-related stress and depressive symptoms varied under different level of perceived social support. Simple slope analysis was conducted to explore the moderation effect further. The interaction plot based on values plus and minus one standard deviation from the mean of the moderator showed the strength of the slopes [45]. In order to avoid multicollinearity, continuous variables were all centralized before the model was validated. Categorical variables were dummy coded, as necessary, for inclusion in multiple regression procedures. Level of significance was set at 0.05 in the study.

## Results

### Descriptive Statistics

The descriptive statistics of the sample are shown in Table 1. The variables included were PHQ-9, gender, educational background, current place of residence, residence style, outbreak of Covid-19 in the city, age, Covid-19 pandemic-related stress, coping style, and PSSS. In general, 51 (9.83%), 16 (3.08%) and 11 (2.12%) students had mild, moderate and severe depressive symptoms, respectively.

**Table 1:** Descriptive statistics

Variables		N (%)	Mean ± SD
PHQ-9	No depressive symptom	441 (84.97)	-
	Mild depressive symptoms	51 (9.83)	-
	Moderate depressive symptoms	16 (3.08)	-
	Severe depressive symptoms	11 (2.12)	-
Gender	Male	276 (53.18)	-
	Female	243 (46.82)	-
Educational background	Undergraduate	453 (87.28)	-
	Master’s	32 (6.17)	-
	Doctoral	27 (5.20)	-
	Trainees	7 (1.35)	-
Current place of residence	China	68 (13.10)	-
	Asia outside China	376 (72.45)	-
	Other continents	75 (14.45)	-
Residence style	Live with family or friends	349 (67.24)	-
	Live alone	170 (32.76)	-
Outbreak in the city	No	110 (21.19)	-
	Yes	359 (69.17)	-
	Do not know the actual situation	50 (9.64)	-
Age		-	22.76 ± 3.60
Covid-19 pandemic-related stress		-	11.95 ± 3.05
Coping style	Positive coping	-	1.61 ± 0.73
	Negative coping	-	1.32 ± 0.70
PSSS		-	60.78 ± 17.64

### Correlations among Variables

The correlations among continuous variables are shown in Table 2. Results revealed that age, Covid-19 pandemic-related stress, negative coping and perceived social support were significantly related with PHQ-9 score. However, positive coping was not significantly correlated with PHQ-9 score.

**Table 2:** Correlations among variables

Variable	1	2	3	4	5	6
1. Age						
2. Covid-19 pandemic-related stress	-0.02					
3. Positive coping	-0.03	0.01				
4. Negative coping	-0.06	0.16^**^	0.51^**^			
5. PSSS	0.04	-0.09^*^	0.33^**^	0.17^**^		
6. PHQ-9	-0.12^*^	0.35^**^	0.02	0.27^**^	-0.24^**^	

Notes: ^*^ P < 0.05, ^**^ P < 0.01

### Differences of Depressive Symptoms in Categorical Variables

The differences of PHQ-9 score in categorical variables are presented in Table 3. The differences were found in residence style and the variable outbreak in the city. The PHQ-9 score was lower when the students lived with family or friends. According to the results of comparison between groups of outbreak in the city, the students who stayed in the cities without Covid-19 outbreak had a lower PHQ-9 score than the students who did not know the actual situation of outbreak or stayed in the cities with Coivid-19 outbreak.

**Table 3:** Differences of PHQ-9 scores in categorical variables

Variables		Median	Mann-whitney U/ Kruskal-Wallis H
Gender	Male	2	33,403
	Female	2	
Educational background	Undergraduate	2	7.75
	Master’s	0	
	Doctoral	2	
	Trainees	1	
Current place of residence	China	1.5	3.15
	Asia outside China	2	
	Other continents	1	
Residence style	Live with family or friends	2	25,680^*^
	Live alone	3	
Outbreak in the city	No	1^#△^	11.46^**^
	Yes	2	
	Do not know the actual situation	3	

Notes: ^*^ P < 0.05, ^**^ P < 0.01; ^#^ P < 0.01 compared with yes, ^△^ P < 0.01 compared with do not know the actual situation

### Results of the Simple Mediation Model Testing

Since positive coping was not significantly correlated with depressive symptoms, only negative coping was introduced into the simple mediation model as a mediator. Results of the simple mediation model testing are shown in Table 4. After controlling for age, residence style and outbreak in the city which were significantly related to depressive symptoms in the univariate analyses, Covid-19 pandemic-related stress was directly associated with PHQ-9 score [coefficient=0.51, 95% confidence interval: (0.37, 0.66)]. A significant indirect effect of Covid-19 pandemic-related stress on PHQ-9 score via negative coping was also found [coefficient=0.07; 95% confidence interval: (0.03, 0.11)]. As the direct effect and the indirect effect were both significant, negative coping partially mediated the association between Covid-19 pandemic-related stress and depressive symptoms.

**Table 4:** Results of the simple mediation model testing

Effect	Coefficient	SE	t	LLCI	ULCI
Direct effect	0.51	0.07	6.89^**^	0.37	0.66
Indirect effect	0.07	0.02	-	0.03	0.11

Notes: SE, standard error; LLCI, lower level of the confidence interval; ULCI, upper level of the confidence interval; ^**^ P < 0.01

### Results of the Moderated Mediation Model Testing

Moderated mediation analysis was performed to assess whether depressive symptoms were indirectly affected by Covid-19 pandemic-related stress via mediation through negative coping and whether the effect was conditionally moderated by perceived social support. Table 5 presents the results of the moderated mediation model. Age was negatively correlated with PHQ-9 score (β=-0.08, t=-2.16, P < 0.05). The PHQ-9 score was lower when the students lived with family or friends (β=-0.12, t=-3.00, P < 0.01). The students who did not know the actual situation of outbreak had a higher PHQ-9 score than the students who stayed in the cities without Covid-19 outbreak (β = 0.10, t = 2.31, P < 0.05). The associations between Covid-19 pandemic-related stress (β = 0.27, t = 6.87, P < 0.01), negative coping (β = 0.26, t = 6.60, P < 0.01) and PHQ-9 score were also significant. Perceived social support had a negative association with PHQ-9 score (β=-0.26, t=-6.25, P < 0.01). The effect of the interaction between negative coping and perceived social support on PHQ-9 score was statistically significant (β=-0.21, t=-5.23, P < 0.01). The variables above accounted for 28% for the variance in the model.

**Table 5:** Results of the moderated mediation model testing

Variable	β	SE	t	LLCI	ULCI
Age	-0.08	0.06	-2.16^*^	-0.24	-0.01
Residence style (Live with family or friends vs. Live alone)	-0.12	0.45	-3.00^**^	-2.25	-0.47
Outbreak in the city					
Yes vs. No	0.02	0.54	0.45	-0.82	1.31
Do not know the actual situation vs. No	0.10	0.82	2.31^*^	0.29	3.52
Covid-19 pandemic-related stress	0.27	0.07	6.87^**^	0.35	0.63
Negative coping	0.26	0.31	6.60^**^	1.44	2.66
PSSS	-0.26	0.01	-6.25^**^	-0.11	-0.06
Negative coping × PSSS	-0.21	0.01	-5.23^**^	-0.10	-0.05
F	24.22^**^				
R^2^	0.28				

Notes: *β *standardized coefficient; *SE *standard error; *LLCI *lower level of the confidence interval; *ULCI *upper level of the confidence interval; ^*^ *P* < 0.05, ^**^ *P* < 0.01

**Fig. 2:**
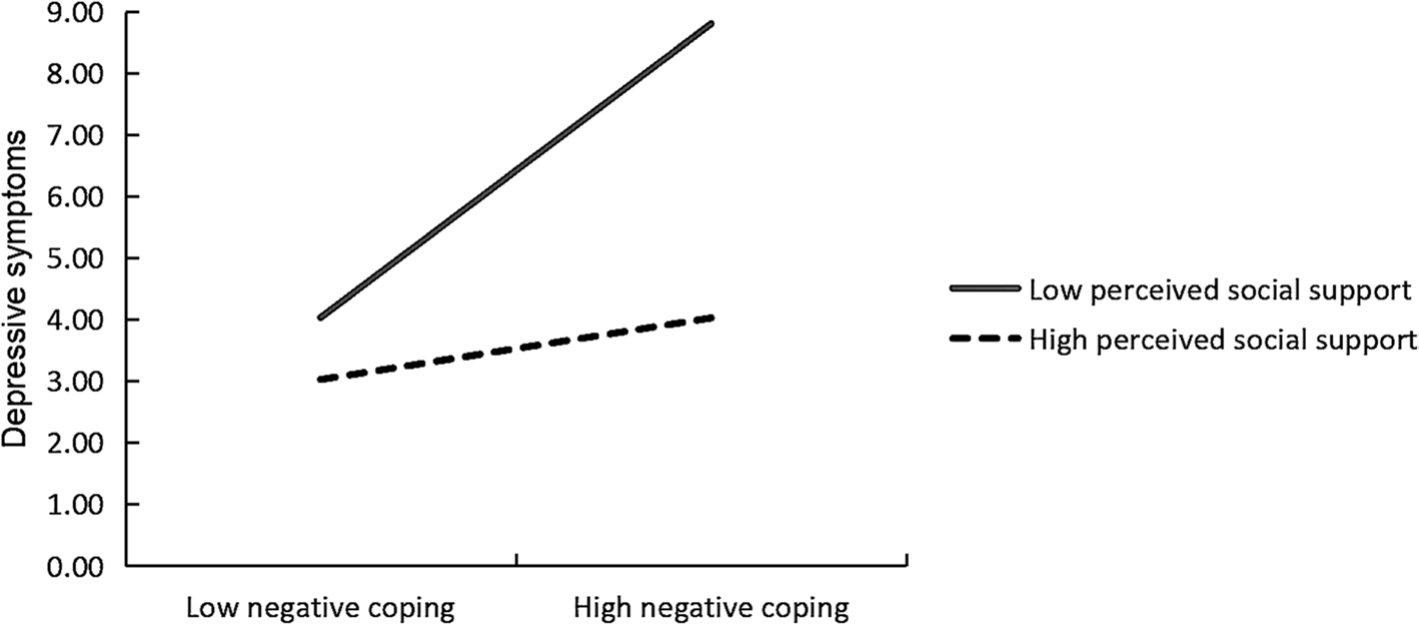
The moderating effect of perceived social support on the relationship between negative coping and depressive symptoms

The nature of the interaction was examined further by the simple slope analysis, and the results were depicted in Fig. 2. As shown in Fig. 2, the plot displayed that negative coping was positively correlated with PHQ-9 score when the level of perceived social support was low (simple slope=3.39, t = 8.67, p < 0.01), while the association was not significant when the level of perceived social support was high (simple slope=0.71, t = 1.72, p = 0.09).

The conditional indirect effect of Covid-19 pandemic-related stress on depressive symptoms through negative coping at various values of perceived social support was analyzed when the score of PSSS was the sample mean and at plus or minus one standard deviation. The results are shown in Table 6. The conditional indirect effect was significant at a lower level and at the mean of PSSS, but not at a higher level of PSSS. These results revealed the indirect effect of Covid-19 pandemic-related stress on depressive symptoms through negative coping weakened as the level of perceived social support increased.

**Table 6:** Conditional indirect effects of Covid-19 pandemic-related stress on PHQ-9 score at different levels of perceived social support

Level of PSSS	Indirect effect	SE	LLCI	ULCI	Index of moderated mediation (95%CI)
-1SD	0.12	0.04	0.06	0.21	-0.003 (-0.005, -0.001)
Mean	0.08	0.02	0.03	0.13	
+1SD	0.03	0.02	-0.01	0.08	

Notes: *β *standardized coefficient; *SE *standard error; *LLCI *lower level of the confidence interval; *ULCI *upper level of the confidence interval

## Discussion

### Depressive Symptoms among International Medical Students

Depression in the students is one of the top concerns for university administrators and educators worldwide. Reported rates of depression or depressive symptoms among college students from various sources in the past generally ranged from 13 to 66.8% [46–50]. In our study, we found that 9.83%, 3.08% and 2.12% students had mild, moderate and severe depressive symptoms, respectively, during the Covid-19 pandemic.

### The Association between Covid-19 Pandemic-related Stress and Depressive Symptoms

Previous researches showed that the outbreak of infectious diseases had adverse effects on students’ mental health [51]. In our study, we also found a significant positive association between Covid-19 pandemic-related stress and depressive symptoms among international students (β = 0.27, P < 0.01), which supported our first hypothesis. Furthermore, the standardized coefficient of Covid-19 pandemic-related stress was the largest among the variables in the model, indicating that stress had the strongest association with the depressive symptoms. The finding was also consistent with those of other studies [52, 53], which provided evidence that Covid-19 pandemic-related stress adversely impacts on depression.

### The Mediating Effect of Coping

Based on the stress coping theory proposed by Lazarus and colleagues, we tested the simple mediation model which hypothesized (H2) that the coping played a mediating role in the association between Covid-19 pandemic-related stress and depressive symptoms among international medical students. The results supported H2 partially, because the mediation effect was only found in the association via negative coping, not via positive coping. This finding indicated that the international students who perceived more Covid-19 pandemic-related stress tended to adopt more negative coping. This phenomenon deserves attention, because it may lead to more subsequent depressive symptoms. In support of our views, it has been found that depressive individuals in stressful situations used avoidant coping strategies more often and had more difficulties in observing positive aspects of stressful life events [54]. Moreover, the use of avoidant coping strategies was found related to higher levels of depressive symptoms [55]. Similarly, results from the university students showed that the more frequently the passive coping strategies were used, and more severe depressive symptoms were reported among the overseas Chinese students who were more likely to choose the passive coping strategies under stressful situations [16]. Moreover, Lardier Jr et al. found that higher level of stress among Hispanic college students was related to higher level of reactive and suppressive coping strategies, which in turn was associated with more depressive symptoms [14]. Thus, reduction of adopting the negative coping style may indirectly reduce the adverse effect of Covid-19 pandemic-related stress on depressive symptoms among international students.

### The Moderating Effect of Perceived Social Support

In the moderated mediation model we proposed (H3) that perceived social support would play a moderating role in the association between negative coping and depressive symptoms among international students. Our results confirmed H3 in that the effect of the interaction between negative coping and perceived social support on depressive symptoms was statistically significant. Further simple slope analysis revealed that negative coping was positively correlated with depressive symptoms only when perceived social support was low, which indicated that international students who chose more negative coping would have more severe depressive symptoms when they perceived less social support. Results also showed that when the level of perceived social support was high, there wouldn’t be more depressive symptoms due to the beneficial effect of social support on mental health. This finding is in accordance with previous research highlighting the moderating role of perceived social support on individual’s depressive symptoms [28, 56]. A possible explanation is that when the international students adopt more negative coping strategies, social relationships may alleviate the adverse impact by providing improvement of students’ self-efficacy, which leads to more encouragement and courage to decrease depressive symptoms [57].

With regard to the conditional indirect effects of Covid-19 pandemic-related stress on depressive symptoms through negative coping at different values of perceived social support, our results indicated a weakened indirect effect with the increase of perceived social support, which supported H4. When the level of perceived social support was low, depressive symptoms were affected more by negative coping style, thus the impact of Covid-19 pandemic-related stress might be stronger. The finding implied the positive role of perceived social support on reducing the indirect adverse effect of Covid-19 pandemic-related stress on depressive symptoms via negative coping. Besides, we also found a significant negative association between perceived social support and depressive symptoms among international students, which also supported the main effect of social support on mental health. In brief, these findings confirmed that perceived social support was a significant protective factor for depressive symptoms among international students, which were in line with results from other studies [58, 59].

### The Association between Demographic Variables and Depressive Symptoms

Among the demographic variables controlled in the final model, age was negatively correlated with depressive symptoms among international students. The possible reason is that elder students have more knowledge and skills to deal with diseases. Therefore, they may have more stable psychological status compared with younger students. Living with family or friends was also a protective factor for depression in our study. According to the research on stress from social groups, the threat and negative influence of stressful situations will be greatly reduced if people shared the situations with others [60]. If the individual is in a totally unsupported environment to encounter life events, he or she has to cope with the stressful situations all alone, and they will feel more threatened, which may lead to a negative impact on mental health. In addition, our research revealed that the students who did not know the actual situation of Covid-19 outbreak had more depressive symptoms than the students who stayed in the cities without outbreak. This may not be surprising since the uncertainty to the outbreak is also a stressful state for the international students.

### Limitations

This study has a few limitations. Firstly, as the participants were all from a medical university, so the results may not apply equally well to the students from other type universities. Secondly, all measures used in this study were self-reported, so there may be discrepancies between what the students reported and how they actually performed. Thirdly, the study used cross-sectional data, so a longitudinal model in the future is needed to verify the causalities among the variables. Fourthly, although this study was designed to examine the association between Covid-19 pandemic-related stress and depressive symptoms, it is possible that other variables also influenced the results of this investigation.

Notwithstanding these limitations, this study supports the stress coping theory by providing evidence of mediation effect of coping in the association between Covid-19 pandemic-related stress and depressive symptoms. In addition, the current study supports the beneficial role of perceived social support on mental health by finding the negative moderation effect of perceived social support on the relationship between negative coping and depressive symptoms, as well as on the indirect effect of Covid-19 pandemic-related stress on depressive symptoms via negative coping. Finally, the findings of the present study have important implications for university educators and administrators. As Covid-19 pandemic-related stress is positively associated with students’ mental health and the pandemic is still going on in many parts of the world, protective intervention or prevention is urgently needed. University educators, administrators or even the students themselves should be educated to identify the risk or protective factors of mental health and understand the interaction mechanism among these variables. In this regard, findings of this study should be very conducive for the university staff and students to combat the adverse mental health outcomes of the pandemic, because several risk or protective factors of depressive symptoms were identified and the mechanism of interaction revealed. What is more important, most of these factors are modifiable so that we are provided with many opportunities or potentials to protect or improve the students’ mental health during the difficult times.

## Conclusions

In this study, we carried out a research on stress and depressive symptoms among international medical students during the Covid-19 pandemic. We found a positive association between the Covid-19 pandemic-related stress and the depressive symptoms, and that negative coping mediated the association. In addition, we found that perceived social support played a moderating role in the relationship between negative coping and depressive symptoms, and moderated the indirect effect of Covid-19 pandemic-related stress on depressive symptoms via negative coping. Findings from the current study highlighted the need for reducing the negative coping and increasing the perceived social support among international medical students under the background of continuing pandemic, as this may help international medical students alleviate the depressive symptoms induced by Covid-19 pandemic-related stress. Training programs for that purpose should be recommended to implement within the campus to reduce the depressive symptoms of international medical students in China.

## Data Availability

The data used during the study are available from the corresponding author on reasonable request.
